# Endless Single-Mode Photonics Crystal Fiber Metalens for Broadband and Efficient Focusing in Near-Infrared Range

**DOI:** 10.3390/mi12020219

**Published:** 2021-02-21

**Authors:** Qiancheng Zhao, Jiaqi Qu, Gangding Peng, Changyuan Yu

**Affiliations:** 1 Photonics Research Center, Department of Electronic and Information Engineering, The Hong Kong Polytechnic University, Hong Kong 999077, China; qiancheng.zhaol@polyu.edu.hk (Q.Z.); jiaqi.qu@connect.polyu.hk (J.Q.); 2 Photonics & Optical Communication, School of Electrical Engineering, University of New South Wales, Sydney, NSW 2052, Australia; g.peng@unsw.edu.au

**Keywords:** optical fiber, metalens, fiber-integrated device, broadband, focusing efficiency

## Abstract

The advent of the ‘lab-on-fiber’ concept has boosted the prosperity of optical fiber-based platforms integrated with nanostructured metasurface technology which are capable of controlling the light at the nanoscale for multifunctional applications. Here, we propose an endless single-mode large-mode-area photonic crystal fiber (LMA-PCF) integrated metalens for broadband and efficient focusing from 800 to 1550 nm. In the present work, the optical properties of the substrate LMA-PCF were investigated, and the metalens, consisting of dielectric TiO_2_ nanorods with varying radii, was elaborately designed in the fiber core region with a diameter of 48 μm to cover the required phase profile for efficient focusing with a high transmission. The focusing characteristics of the designed metalens were also investigated in detail over a wide wavelength range. It is shown that the in-fiber metalens is capable of converging the incident beams into the bright, symmetric, and legible focal spots with a large focal length of 315–380 μm depending on the operating wavelength. A high and average focusing efficiency of 70% was also obtained with varying wavelengths. It is believed the proposed fiber metalens may show great potential in applications including fiber laser configuration, machining, and fiber communication.

## 1. Introduction

Since the first demonstration of silica optical fiber with a low transmission loss of 20 dB/km in the early 1970s [1], low-loss optical fibers have revolutionized the telecommunication industry in the last five decades and have continued to evolve toward an extremely low level with a value of 0.14 dB/km at 1550 nm [2]. To date, various types of optical fibers have been developed and serve as a well-established platform for efficient light guiding and transmission. A large number of applications based on optical fiber technology have been realized, such as optical sensing for various physical parameters [3,4], fiber lasers and amplifiers [5,6], and astrophysics [7]. Despite the tremendous success of optical fiber technology, there exist some drawbacks hindering the widespread application of optical fibers. The optical properties of optical fibers (amplitude, phase, polarization, etc.) are difficult to alter once they are fabricated. Moreover, the transmitted modes in the fiber core tend to diverge which are restricted by the diffraction limit of the core size. As a result, extensive research has been conducted into the direction of fiber-integrated devices. A variety of functionalized fiber-based devices, such as plasmonic sensors [8], biosensors [9,10,11], and ultrafast fiber lasers [12,13], have been successfully fabricated by implanting periodic nanostructures on the facets of the optical fibers. Particularly, in-fiber focusing lenses have also been demonstrated in recent years, with the specially designed plasmonic concentric annulus on the fiber end facet [14,15]. However, these optical devices operate in a very short focal length, and their focusing performance was strongly affected by high-order diffraction patterns, limiting their potential in a range of optical applications.

Fortunately, the emergence of metasurface technology has attracted enormous attention due to its superior features in tuning the properties of the incident light wavefront by controlling the phase polarization and amplitude with the spatially designed flat and periodic arrays in subwavelength scale. Until now, a large number of optical meta-devices have been developed with multiple functions including beam splitter [16,17] and light-field imaging [18,19,20]. The use of fiber-integrated meta-devices, with the ‘lab-on-fiber’ paradigm, has also recently been demonstrated in applications such as an in-fiber polarizer [21], scanning microscopy [22,23], and laser mode-locking [24]. In particular, the in-fiber plane metalens with the focusing effect has also been reported recently [25]. However, the focusing function was polarization-dependent with a focusing efficiency of only 16% due to metal loss. Moreover, the focal length was only several tens of micrometers with a narrow operating bandwidth of 100 nm, which limits its practical application. In this regard, we propose an endless single-mode photonic crystal fiber (PCF) integrated metalens which operates in a broadband wavelength range from 800 to 1550 nm. The carefully designed in-fiber metalens is composed of spatially arranged dielectric titanium dioxide (TiO_2_) with varying diameters and provides sufficient phase coverage for efficient focusing of fiber guided modes with high efficiency of 70%. We believe that the proposed large-mode-area PCF metalens show great potential in a number of in-fiber applications such as fiber laser configuration, fiber sensing, and fiber communication. 

## 2. Materials and Methods 

### 2.1. Design of Endless Single-Mode LMA-PCF

**Figure 1:**
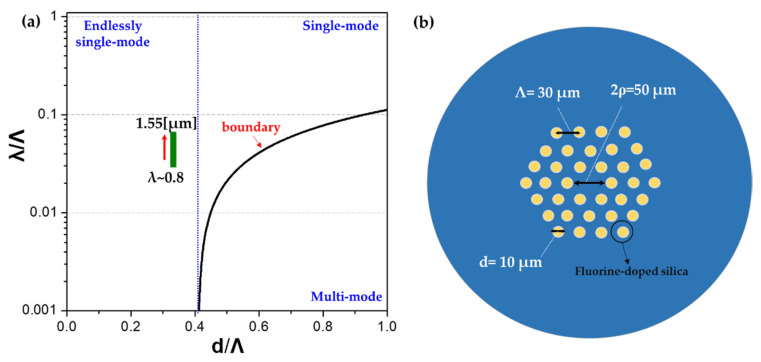
(**a**) Simulated curve for single-mode and multi-mode boundaries of designed photonic crystal fibers (PCFs). (**b**) Geometry and key parameters of the designed all-glass PCF.

To select the substrate for loading of the multifunctional metalens array, one of the key criteria is to ensure a large core mode area to place a large number of nano units for sufficient modulation of phase. In this regard, photonic crystal fibers (PCFs) are one of the most efficient media where the endless single-mode propagation can be achieved by the special geometry arrangement of cladding and core. In this work, instead of the conventional air-hole PCF structure in which precisely controlling the dimension of air holes in fiber fabrication is quite demanding and labor-intensive, the all-glass PCF structure is proposed where the air holes are replaced by the commercially available fluorine-doped silica rods, similar to the ones reported in [26,27]. The core and cladding have an extremely low refractive index difference of Δ*n* ~1.2 × 10^−3^. Theoretically and experimentally [28,29], for a well-constructed PCF, the normalized frequency parameter *V* can be written as

(1) $$V_{\mathrm{PCF}}=\frac{2\pi \Lambda }{\lambda }{\left[n_{c}^{2}\left(\lambda \right){-n}_{\mathrm{cl}}^{2}\left(\lambda \right)\right]}^{\frac{1}{2}}$$

where *n*_c_(*λ*) and *n*_c_(*λ*) represents the refractive index of the core and cladding at a given wavelength, respectively. *Λ* is the lattice constant, namely the pitch between the hole spacing. For single-mode guiding of a standardized PCF, the *V*_PCF_ number should be controlled as *V*_PCF_ ≤ π. Taking into the refractive index difference from our design, the ratio of incident wavelength to the pitch (*λ*/*Λ*) should be set above ~0.117. On the other hand, from the multipole solutions of Maxwell’s equations, it has been found the phase boundary distinguishing the single from the multimode propagation is well fitted by the following equation [30]:

(2) $$\frac{\lambda }{\Lambda }=\alpha {\left[\frac{d}{\Lambda }-\frac{d*}{\Lambda }\right]}^{\gamma }$$

where the d*/*Λ* is the empirical critical parameter (~0.406) for standardized PCF. Considering the critical value of λ*/*Λ* (~0.117) and the optimal fitting with *α* = 0.18, *γ* = 0.89, the phase diagram illustrating the single-mode and multi-mode regimes of the designed all-glass PCF is plotted, as shown in Figure 1a. As can be seen, for the region *d*/*Λ* < d*/*Λ*, the PCF has the endless single-mode propagation. For *d*/*Λ* > d*/*Λ* and *λ*/*Λ* > λ*/*Λ*, the single-mode transmission is still supported whereas the second-order mode is confined in the core where *λ*/*Λ* < λ*/*Λ*. 

**Figure 2:**
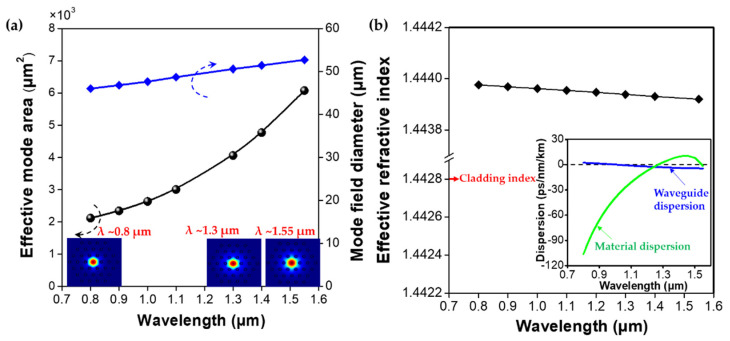
(**a**) Effective mode area and mode field diameter as a function of the operating wavelength. (**b**) Effective refractive index versus operating wavelength. The inset shows the wavelength dependences of different dispersion curves.

Following the phase diagram illustrated in Figure 1a, we have configured the all-glass endless single-mode PCF, as indicated by the green rectangle. The all-glass PCF is designed with *Λ* = 30 μm, *d* = 10 μm, and core diameter of 2*ρ* = 50 μm, as shown in (see Figure 1b). It is worth noting that the core area of the designed PCF is approximately 1.6 times larger than the commercially available large-mode-area-25 (LMA-25) PCF. The operating wavelength is then implemented from 800 to 1550 μm to cover three typical fiber communication windows. Firstly, the transmission properties of the designed PCF are analyzed and shown in Figure 2a. It is seen that with the increase in the operating wavelength, both the effective mode area and the mode field diameter (MFD) of the fundamental mode increase monotonically and reach the maximum at 1550 nm. It is worth noting that the mode area for the designed fiber at 1550 μm has been enlarged nearly 60 times compared to the Corning single-mode fiber 28 (SMF-28), making it suitable for the application of fiber amplifiers and lasers because of its capability of loading high power levels with the ease of nonlinear limits. The wavelength dependence of the effective refractive index of the fundamental mode is also plotted in Figure 2b. As expected, the effective refractive index of the guided mode decreases with the increase in wavelength. The dispersion properties of the designed PCF are also illustrated in the inset of Figure 2b. It is seen that the material dispersion dominates the waveguide dispersion in the desired spectral range, and the waveguide dispersion is negligibly small for this kind of LMA-PCF.

### 2.2. Design of Broadband Focusing Metalens 

**Figure 3:**
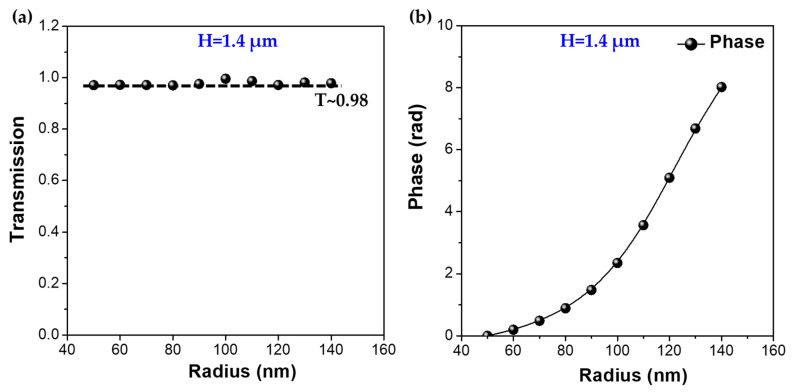
(**a**) The transmission as a function of the nanorod radius at the height of *H* = 1.4 μm. (**b**) The phase delay as a function of the nano function of the nanorod radius at the height of *H* = 1.4 μm.

After careful design of the endless single-mode LMA-PCF as the substrate, the metalens array was also designed based on the core region of the LMA-PCF. In general, there are two kinds of phase modulation that would lead to the convergence of incident light beam, namely the propagation phase and geometry phase. The geometry phase, which is also called the Pancharatnam–Berry (PB) phase, originates from the rotation (i.e., rotation angle *θ*) of the nano unit through which the cross-polarized beam carries an additional phase change of 2*θ* [31].By contrast, the propagation phase is modulated by the size (i.e., height, radius) of each unit cell. As a result, the propagation phase covering from 0 to 2π can be achieved by continuously tuning the dimension of the unit cell along a certain orientation, say, the *x* axis. By using this method, the desired optical path difference is attained by changing the optical path of the light, which is independent light polarization. Theoretically, to achieve the focusing effect for the incident beam, the phase profile of the designed metalens should satisfy the following equation [32]:

(3) $$\varphi (x,\;y)=\frac{2\pi }{\lambda }(\sqrt{f^{2}{+x}^{2}{+y}^{2}}-f)$$

where *f* is the designed focal length, *λ* is the wavelength of the incident beam, and *x* and *y* are the coordinates in the plane of metalens array. To achieve the 2π phase retardation from the center to the edge of the metalens array, the relationship between the focal length and the radius of metalens can be rewritten as

(4) $$f=\frac{R^{2}}{2\lambda }-\frac{\lambda }{2}$$

where *R* is the radius of the designed metalens. From Equation (4), it is clear that the focal length depends tightly on the radius of the metalens. Therefore, it is apparent that the LMA-PCF supports a larger focal length with a higher focusing efficiency. Here, the dielectric titanium oxide (TiO_2_) nanorod with an effective index of ~2.55 is employed as the unit cell of the metalens for tuning the propagation phase since the loss of TiO_2_ is negligible at the optical communication wavelengths. It should be noted that in addition to the requirement of phase retardation, the transmission level through the nanorods should also be high enough to reduce the transmission loss and allow for high focusing efficiency. In this regard, both the radius and height of the nanorod have to be tailored to fulfill these two requirements. Figure 3a,b illustrate the transmission and the phase change induced by the radius of nanorod with the given optimal height (*H* ~1.4 μm) and period (*Ʌ* ~1 μm) of the nanorods. 

**Figure 4:**
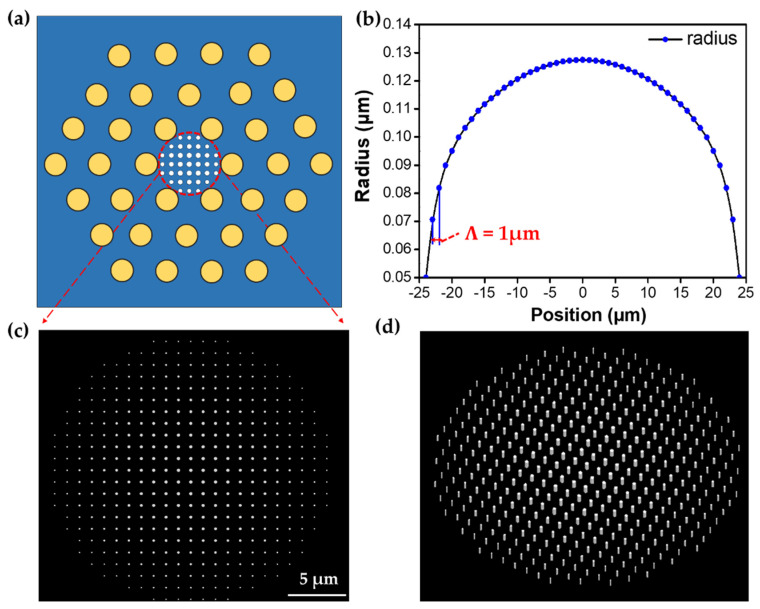
(**a**) Schematic diagram of the dielectric large-mode-area (LMA)-PCF integrated metalens. (**b**) Relationship between the nanorod radius and the spatial position in the metalens. (**c**) Enlarged top view of the nanorod array constituting the metalens in the region of (**a**). (**d**) Perspective view of the elements of the metalens loaded on the LMA-PCF.

It is seen that at the predefined height and period, the phase delay induced by the nanorod increases along with the radius and achieves full 2π phase coverage by varying the radius from 50 to 140 nm, whereas a high transmission coefficient (*T* ~0.98) is maintained for the whole radius range. This suggests that the designed parameters of the metalens on top of the LMA-PCF can substantially act as an efficient focuser which may find great potential in the fiber communication domain. Based on the phase versus radius relation and the target hyperbolic phase versus position relation, the required nanorod radius at a given spatial position is calculated. It should be noted that the diameter of the designed metalens should be smaller than that of the core region of the PCF since the guided mode is tightly confined in the center of the PCF. Taking into account the relationship between radius and position, Equation (4), and the core size of the endless single-mode LMA-PCF, the proposed PCF-based metalens is elaborately constructed for efficient focusing of the broadband wavelength range used in the fiber-optic communication systems, as shown in Figure 4a. It is seen that, with the well-established relation between the nanorod radius and the spatial position (Figure 4b), the diameter of the nanorods decreases radially and orderly from the center to the edge of the metalens (Figure 4c,d), fulfilling the 2π propagation phase change over the radial direction of metalens. Since we are interested in the focusing effect in the short wavelength region, the metalens is primarily designed to converge the incident beam at a short wavelength of 0.8 μm with a lens diameter of 48 μm and a large focal length of 360 nm. 

## 3. Results and Discussion 

**Figure 5:**
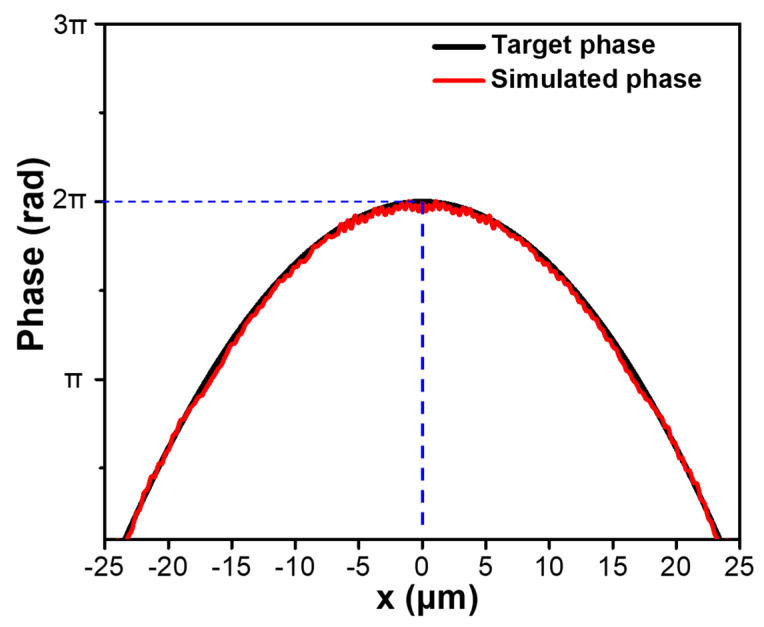
Simulated and target phase profiles along the *x* axis of the designed LMA−PCF metalens.

To verify the focusing effect of the designed metalens, the guided fundamental mode of the LMA-PCF was selected and launched into the metalens to monitor the optical path traveling through it. The numerical simulation was carried out using the finite difference time domain (FDTD) method, and the incident light is assumed to propagate along the +*z* axis. Firstly, the phase retardation of the incident beam across the *x* axis is computed and plotted in Figure 5. 

It is seen that the simulated phase profile well coincides with the required phase retardation, exhibiting 2π variation across the radial direction of the metalens. Still, it should be noted that some ripples are observed in the simulated phase profile which is caused by the discretization using the phase-to-radius mapping with a periodicity of 1 μm, and the precision of phase retardation modulated by the nanorod array is expected to be improved with a shorter periodicity.

**Figure 6:**
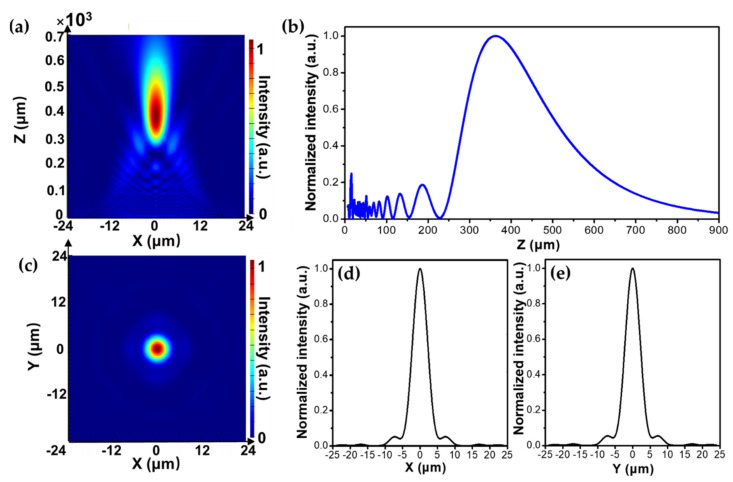
(**a**) Normalized intensity distribution of the focal point at the *x*−*z* plane upon illumination at 800 nm. (**b**) Normalized intensity profile along the *z* axis in (**a**). (**c**) The normalized intensity distribution of the focal spot at the *x*−*y* plane. (**d**,**e**) Normalized intensity profiles of the corresponding focal plane along the *x* and *y* direction, respectively.

To visualize the focal point at a certain distance with the designed in-fiber metalens, the near to far-field projection method was applied with the period matched layer (PML) boundary conditions along the propagation direction (+*z* axis). Figure 6a presents the focusing characteristics of the designed metalens in the propagation (*x*–*z*) plane at the desired wavelength *λ*_d_ = 800 nm. It is seen that the incident beam traveling through the metalens becomes converged and reaches the maximum intensity at a distance of *z* = 380 μm. Moreover, the light intensity profile along the *z*-direction at *x* = 0 is also plotted in Figure 6b. It can be seen that the light intensity firstly oscillates in the region of *z* = 0 to 225 μm, which may be caused by the stray lights which are not tightly focused. Thereafter, the light intensity increases sharply from *z* = 225 to 380 μm and gradually decreases as the light beam propagates farther, confirming that the incident light beam is well focused at a distance of 380 μm. The simulated focal length is similar to the presumed value of 360 μm obtained by Equation (4) with a small discrepancy, which may be attributed to the limited number of nanorods and spatial distance acquired by the discretized phase-to-radius mapping method. In addition, the intensity distribution of the focal spot across the focal (*x*–*y*) plane is presented in Figure 6c. Clearly, a tightly focused, bright, and symmetric focal spot is observed in the center of the focal plane. Furthermore, the normalized intensity profiles of the focal spot along the *x*- and *y*-direction are present in Figure 6d,e. Clearly, the same intensity profile is observed along the two orthogonal directions at the focal plane with the maximal intensity located in the center. This further manifests the central symmetric of the focal spot with the well-focused characteristics of the designed in-fiber metalens. By calculation, the focused beam waist, defined as the full width at half maximum (FWHM), is also obtained with a value of 5 μm at the target wavelength of 800 nm.

**Figure 7:**
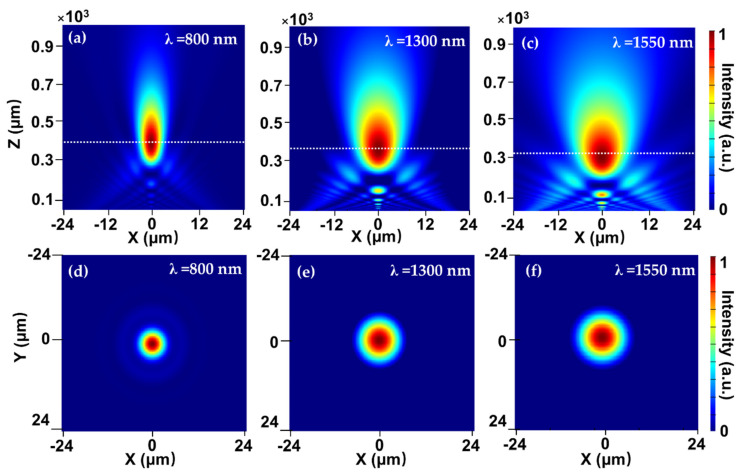
Normalized intensity distribution of the focal spot at the *x*−*z* plane at various incident wavelengths of (**a**) 800 nm, (**b**) 1300 nm, and (**c**) 1550 nm. Normalized intensity distribution of the focal spot at the *x*−*y* plane at incident wavelengths of (**d**) 800 nm, (**e**) 1300 nm, and (**f**) 1550 nm.

In addition to the target wavelength in the short wavelength region, the focusing performance of the in-fiber metalens is also investigated with the second and third telecommunication windows at 1300 and 1550 nm. The far-field intensity distribution of the in-fiber metalens in the *x*–*z* plane at various incident wavelengths is shown in Figure 7a–c. It is seen that with the increase of operating wavelength, the focal length is moderately decreased, which is in agreement with the relationship shown in Equation (4). However, the focal region is seen to be notably enlarged at longer wavelengths; this is because the 2π phase coverage is designed for the target wavelength of 800 nm and less phase coverage is achieved across the metalens for longer wavelengths, resulting in a weaker focusing effect as compared to the optimized wavelength. The intensity distribution of the focal spot in the *x*–*y* plane at three wavelengths is also illustrated in Figure 7d–f. Correspondingly, the cross-section of the focal spot is increased dramatically with increasing incident wavelength, and the electric field intensity of the focal spot decreases radially and smoothly from the center to the edge, showing the good beam quality of the focal spot.

**Figure 8:**
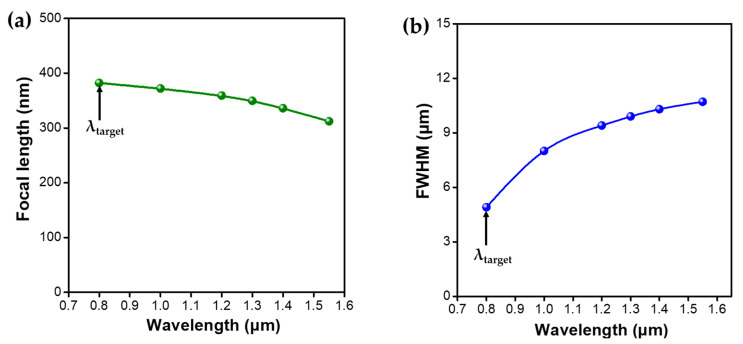
(**a**) Focal length of the designed in-fiber metalens as a function of the operating wavelength. (**b**) The full width at half maximum (FWHM) of the focal spot as a function of the operating wavelength.

To further quantify the focusing performance of the designed in-fiber metalens, the focal length and FWHM of the focal spot at various wavelengths are extracted and calculated, as shown in Figure 8a,b. It can be seen that the focal length has blueshifted from 380 to 310 μm when the incident wavelength is varied from 800 to 1550 nm (Figure 8a), and it shows a decreasing trend with the increase of wavelength. By contrast, the change in FWHM exhibits a reverse trend (Figure 8b), and the value of FWHM is increased monotonically from 5 to 11 μm with the increase of incident wavelength. Based on the optical performance of the designed metalens, it is revealed that the input fundamental mode from the LMA-PCF at all optical communication wavelengths are well focused with the compatible dielectric metalens, manifesting the broadband functionality of this in-fiber meta-device. However, due to the chromatic aberration caused by the different phase accumulation through light propagation with various wavelengths, the potential of the designed in-fiber metalens is limited for full-color optical applications such as long-haul communication, high-precision imaging, and broadband detection. In the near future, achromatic broadband metalens should be created to enable the widespread applications of fiber-integrated meta-devices.

**Figure 9:**
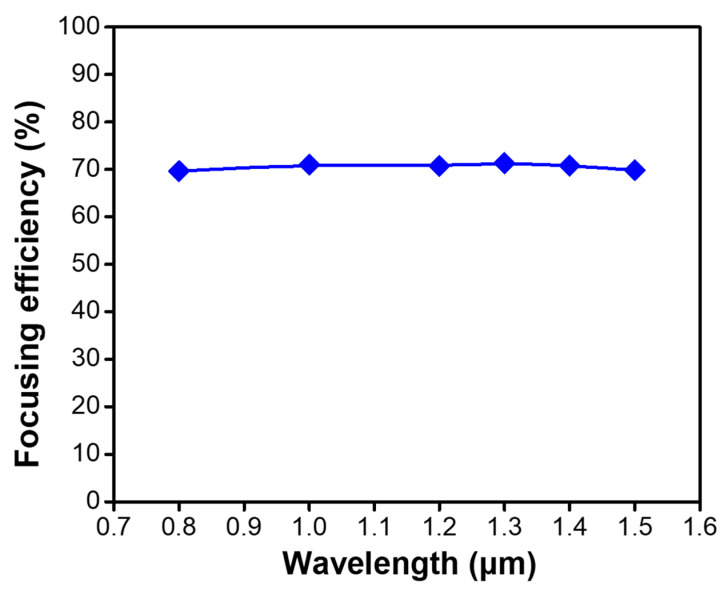
Focusing efficiency of the in-fiber metalens as a function of the operating wavelength.

The focusing efficiency of the in-fiber metalens, which is defined as the ratio of the optical power of the measured focused beam to that of the incident beam [33], has also been investigated at various operating wavelengths from 800 to 1550 nm. The results are shown in Figure 9. It is seen that the focusing efficiency remains negligible change with an average high value of 70% for all the investigated wavelengths, showing steady performance in the broadband wavelength range. The focusing efficiency of the designed in-fiber metalens can be further improved by employing the silicon (Si) nanopillar as the metalens element as Si possesses an even lower transmission loss in the desired transmission wavelength range. The maximal focusing efficiency can be achieved as high as 86% at the incident wavelength of 800 nm.

## 4. Conclusions

In conclusion, this paper has presented an all-dielectric LMA-PCF integrated metalens that operates in a broadband wavelength range from 800 to 1550 nm, including the three typical telecommunication windows. The designed metalens is comprised of periodic circular arrays of high-aspect-ratio TiO_2_ nanopillars, which are placed on a specially designed endless single-mode LMA-PCF. By arranging the radius of the unit cell with the optimal height and periodicity, the sufficient phase coverage and high transmission have been obtained in the target wavelength range. The focusing performance of the designed in-fiber metalens at various incident wavelengths has also been studied. It is found that the in-fiber metalens is capable of tightly focusing various incident lights with legible, symmetric, and bright spots with a large focal length ranging from 310 to 280 μm, and the focusing efficiency is calculated to be as high as 70% and is almost identical for the target wavelength range. It is believed that the proposed fiber-integrated meta-device will pave the way for the creation of miniaturized and multi-functionalized fiber-based devices, which show great potential in a large number of optical applications including in-fiber optical imaging, laser machining, and laser systems.

## Data Availability

Data available upon request from the corresponding author.

## References

[1] Kapron F., Keck D.B., Maurer R.D. (1970). Radiation losses in glass optical waveguides. Appl. Phys. Lett..

[2] Tamura Y., Sakuma H., Morita K., Suzuki M., Yamamoto Y., Shimada K., Honma Y., Sohma K., Fujii T., Hasegawa T. (2018). The First 0.14-dB/km Loss Optical Fiber and its Impact on Submarine Transmission. J. Lightwave Technol..

[3] Xia F., Zhao Y., Hu H.-F., Zhang Y. (2018). Optical fiber sensing technology based on Mach-Zehnder interferometer and orbital angular momentum beam. Appl. Phys. Lett..

[4] Dong S., Dong B., Yu C., Guo Y. (2017). High sensitivity optical fiber curvature sensor based on cascaded fiber interferometer. J. Lightwave Technol..

[5] Jeong Y.e., Sahu J., Payne D.A., Nilsson J. (2004). Ytterbium-doped large-core fiber laser with 1.36 kW continuous-wave output power. Opt. Express.

[6] Wang J., Song Q., Sun Y., Zhao Y., Zhou W., Li D., Xu X., Shen C., Yao W., Wang L. (2019). High-performance Ho: YAG single-crystal fiber laser in-band pumped by a Tm-doped all-fiber laser. Opt. Lett..

[7] Cruz F.C. (2008). Optical frequency combs generated by fourwave mixing in optical fibers for astrophysical spectrometer calibration and metrology. Opt. Express.

[8] Yang X., Ileri N., Larson C.C., Carlson T.C., Britten J.A., Chang A.S., Gu C., Bond T.C. (2012). Nanopillar array on a fiber facet for highly sensitive surface-enhanced Raman scattering. Opt. Express.

[9] Arrizabalaga O., Velasco J., Zubia J., de Ocáriz I.S., Villatoro J. (2019). Miniature interferometric humidity sensor based on an off-center polymer cap onto optical fiber facet. Sens. Actuators B Chem..

[10] Shi X., Ge K., Tong J.-H., Zhai T. (2020). Low-cost biosensors based on a plasmonic random laser on fiber facet. Opt. Express.

[11] Wang B., Deng L., Sun L., Lei Y., Wu N., Wang Y. (2018). Growth of TiO_2_ nanostructures exposed {001} and {110} facets on SiC ultrafine fibers for enhanced gas sensing performance. Sens. Actuators B Chem..

[12] Zhai T., Niu L., Cao F., Tong F., Li S., Wang M., Zhang X. (2017). A RGB random laser on an optical fiber facet. RSC Adv..

[13] Li S., Wang L., Zhai T., Xu Z., Wang Y., Wang J., Zhang X. (2015). Plasmonic random laser on the fiber facet. Opt. Express.

[14] Kim H., Kim J., An H., Lee Y., Lee G.-Y., Na J., Park K., Lee S., Lee S.-Y., Lee B. (2017). Metallic Fresnel zone plate implemented on an optical fiber facet for super-variable focusing of light. Opt. Express.

[15] Liu Y., Xu H., Stief F., Zhitenev N., Yu M. (2011). Far-field superfocusing with an optical fiber based surface plasmonic lens made of nanoscale concentric annular slits. Opt. Express.

[16] Khorasaninejad M., Crozier K.B. (2014). Silicon nanofin grating as a miniature chirality-distinguishing beam-splitter. Nat. Commun..

[17] Khorasaninejad M., Zhu W., Crozier K. (2015). Efficient polarization beam splitter pixels based on a dielectric metasurface. Optica.

[18] Wang S., Wu P.C., Su V.-C., Lai Y.-C., Chu C.H., Chen J.-W., Lu S.-H., Chen J., Xu B., Kuan C.-H. (2017). Broadband achromatic optical metasurface devices. Nat. Commun..

[19] Fan Z.-B., Qiu H.-Y., Zhang H.-L., Pang X.-N., Zhou L.-D., Liu L., Ren H., Wang Q.-H., Dong J.-W. (2019). A broadband achromatic metalens array for integral imaging in the visible. Light Sci. Appl..

[20] Lin R.J., Su V.-C., Wang S., Chen M.K., Chung T.L., Chen Y.H., Kuo H.Y., Chen J.-W., Chen J., Huang Y.-T. (2019). Achromatic metalens array for full-colour light-field imaging. Nat. Nanotechnol..

[21] Wang J., Du J. (2016). Plasmonic and dielectric metasurfaces: Design, fabrication and applications. Appl. Sci..

[22] Mivelle M., Ibrahim I.A., Baida F., Burr G., Nedeljkovic D., Charraut D., Rauch J., Salut R., Grosjean T. (2010). Bowtie nano-aperture as interface between near-fields and a single-mode fiber. Opt. Express.

[23] Mivelle M., van Zanten T.S., Garcia-Parajo M.F. (2014). Hybrid photonic antennas for subnanometer multicolor localization and nanoimaging of single molecules. Nano Lett..

[24] Wang J., Coillet A., Demichel O., Wang Z., Rego D., Bouhelier A., Grelu P., Cluzel B. (2020). Saturable plasmonic metasurfaces for laser mode locking. Light Sci. Appl..

[25] Yang J., Ghimire I., Wu P.C., Gurung S., Arndt C., Tsai D.P., Lee H.W.H. (2019). Photonic crystal fiber metalens. Nanophotonics.

[26] Dong L., McKay H.A., Fu L. (2008). All-glass endless single-mode photonic crystal fibers. Opt. Lett..

[27] Gowre S., Mahapatra S., Varshney S.K., Sahu P.K. (2013). Dispersion characteristics of all-glass photonic crystal fibers. Opt. Int. J. Light Electron Opt..

[28] (2003). Modal cutoff and the V parameter in photonic crystal fibers. Opt. Lett..

[29] (1998). Properties of photonic crystal fiber and the effective index model. J. Opt. Soc. Am. A.

[30] (2002). Modal cutoff in microstructured optical fibers. Opt. Lett..

[31] Wang W., Guo C., Tang J., Zhao Z., Wang J., Sun J., Shen F., Guo K., Guo Z. (2019). High-efficiency and broadband near-infrared bi-functional metasurface based on rotary different-size silicon nanobricks. Nanomaterials.

[32] Aieta F., Genevet P., Kats M.A., Yu N., Blanchard R., Gaburro Z., Capasso F. (2012). Aberration-Free Ultrathin Flat Lenses and Axicons at Telecom Wavelengths Based on Plasmonic Metasurfaces. Nano Lett..

[33] Khorasaninejad M., Zhu A.Y., Roques-Carmes C., Chen W.T., Oh J., Mishra I., Devlin R.C., Capasso F. (2016). Polarization-Insensitive Metalenses at Visible Wavelengths. Nano Lett..

